# Surgeon agreement at the time of handover, a prospective cohort study

**DOI:** 10.1186/s13017-016-0065-6

**Published:** 2016-02-24

**Authors:** Richard Hilsden, Bradley Moffat, Sarah Knowles, Neil Parry, Ken Leslie

**Affiliations:** Department of Surgery, University of Western Ontario, London Health Sciences Centre, University Hospital, 339 Windermere Road, P.O. Box 5339, London, ON N6A 5A5 Canada; Department of Critical Care, London, On Canada; Schulich School of Medicine and Dentistry, Western University, London, ON Canada

**Keywords:** Acute care surgery, Handover, Patient safety

## Abstract

**Background:**

Acute Care Surgical Teams are responsible for emergent surgical patients, and as such require regular handover and coordination between different surgeons. Despite the recent emergence of this model of care, minimal research has been conducted on the quality of patient handover and no research has attempted to determine the rate of clinical agreement or disagreement among surgeons participating in these teams.

**Methods:**

A prospective cohort study was carried out with our acute care surgical service at a tertiary care teaching hospital from January 2 to March 31 2012. At the conclusion of the daily morning handover, receiving surgeons were asked to indicate, on provided handover sheets, whether they agreed with the proposed management plan for each patient that was discussed. The specific aspects of care over which they disagreed were also described, and disagreements were classified a priori as major or minor. The primary outcome was the rate of disagreement over the handed over management plan.

**Results:**

Six staff surgeons agreed to participate and a total of 417 unique patients were handed over during the study period. For the primary outcome, a total of 41 disagreements were recorded for a disagreement rate of 9.8 %. 15 of the 41 disagreements were classified as major, for a major disagreement rate of 3.6 %. Consultant to consultant disagreements were classified as major disagreements 63 % of the time, whereas consultant to resident disagreements were classified as major 31 % of the time (*P* = 0.217). On average, the age of patients for which a clinical disagreement occurred were older; 63 vs. 57 (*P* < 0.05).

**Conclusions:**

Despite the frequency of handovers in clinical practice, little research has been conducted to determine the rate of disagreement over patient management among surgeons participating working in academic centers. This study demonstrated that the rate of clinical disagreement is low among surgeons working in an tertiary care teaching hospital.

## Background

Acute care surgical teams represent an emerging model of surgical care in large hospitals and teaching institutions. These teams are tasked with the specific mandate to care for patients with urgent surgical issues. In high-volume hospitals, the acute care surgical service is frequently called upon by emergency departments, trauma services, and intensive care units [1, 2]. These teams differ from the traditional emergency surgical model: where a single surgeon was responsible for all acute general surgical emergencies over a period of 12–24 h, while simultaneously managing busy elective surgical lists and outpatient responsibilities [3]. The main weakness of this traditional model is the inherent conflict of responsibilities that occurs between the surgeon’s elective and emergency patients [4, 5].

Acute care surgical teams afford several advantages over the traditional model of emergency surgical care in that they allow for clear lines of responsibility to be established in the treatment of emergent surgical patients; they ensure that hospital resources are consistently being allocated to the sickest patients in a timely fashion without drawing focus away from elective patients [6]. At the same time, the decision to shift from the traditional model of surgical care to an acute care surgical model places significant time demands on clinical staff. To manage these demands, regular handover from staff surgeon to staff surgeon is required [1]. As a result, those who administrate acute care surgical services have made continuity of care a priority for these teams [3]. In this context, an appropriate definition of “continuity of care” is the degree to which a patient’s management proceeds along the same care trajectory from admission to discharge [7]. For continuity of care to be maintained throughout the handover process, information must be adequately passed from one party to another, agreement over the patients’ care plans must be met between the incoming and outgoing clinicians, and finally the care plan must ultimately be followed [7].

Until now, research in the realm of patient handover has focused mainly on the frequency and quality of communication during handovers [8–10]. On this issue, an evaluation of sentinel events leading to litigation by the Joint Commission on Accreditation of Healthcare Organization demonstrated that miscommunication of one form or another could be identified in nearly 70 % of medical misadventure cases [11]. Of these, miscommunication was considered to be contributory to a negative outcome in 49 % of cases [11].

It has been shown that over the course of several unique patient encounters different physicians often arrive at different clinical conclusions [12, 13]. Despite this, the medical literature on continuity of care has not addressed the role of clinical agreement in shared health care models. In particular, little research has been done to outline the degree to which surgeons disagree over specific patient management decisions, and no research has been conducted to determine the frequency of concurrence among surgeons during patient handover in a teaching hospital. The primary objective of this study was to determine the frequency of agreement at the time of patient handover among surgeons participating in the acute care surgery team at an academic tertiary care hospital.

## Methods

A prospective cohort design was used to evaluate the rate of clinical agreement by the receiving surgeon regarding the patient management plan at the time of handover. Local institutional ethics board approval was obtained. The surgeons who participate in the general surgery acute care surgical service (ACCESS) at Victoria Hospital, London Health Sciences Centre in London, Ontario were approached via email, and in person (by KL, RH and NP) inviting them to participate. All surgeons who were approached agreed to participate in this study. Surgeons who had privileges at the institution but did not regularly participate as members of the acute care surgery team were excluded. The study period ran from January 9^th^ 2012 to March 24^th^ 2012. During this period there were six different consultant surgeons who covered ACCESS.

The acute care surgical service at our institution consists of a single consultant who leads a team of house staff and is responsible for daytime emergencies from Monday to Sunday. During weeknights, different surgeons from the call pool are responsible for emergency care. On a daily basis, the post-call staff surgeon and house staff meet face to face to hand over all of the overnight surgical patients to the daytime team. The acute care surgery consultant changes on a weekly basis with a face-to-face handover occurring on Monday mornings. All patients on the ACCESS team are discussed during these daily handover sessions. During these meetings patient histories, physical exam findings, investigation results, and patient care plans are discussed in detail between the receiving team and the providers handing off. The receiving consultants are encouraged to question the handing over team members to ensure understanding of all active clinical issues and to clarify the management plans previously set in place. The purpose of these daily handover meetings is to clearly communicate patient care plans and maintain continuity of care as the clinical providers for these patients.

As part of routine care in this institution, the patient handover lists are generated from the electronic medical record so that all the participants in the handover process can discuss patient issues. These patient lists contain names, basic demographics such as gender and age, as well as an admission diagnosis, and are standardized for all patients. These handover sheets provide a platform for the discussion of patient care plans; however, all clinical information is communicated verbally face to face between clinicians participating in the acute care surgery handover.

On a daily basis, the receiving surgeon was given a second copy of the ACCESS patient list upon which he/she was asked to indicate whether he/she agreed or disagreed with the previously established patient management plan. The surgeons were instructed to keep their agreement decisions and all study patient lists confidential. For each patient, where the receiving surgeon felt there was a disagreement, that surgeon was asked to indicate (in point form on the provided handover sheets) the aspects of patient care upon which he/she disagreed. As residents at this institution are given graduated level of responsibility for patient care, the receiving consultants at handover were asked to indicate whether they felt the management plan for which they disagreed originated with the resident or previous consultant. Participating surgeons were encouraged to indicate a disagreement for any situation where they felt their opinion differed over a specific issue, even if they felt the issue was trivial. Participating surgeons were blinded as to how disagreements would be classified in the final analysis.

Each day, following handover, the patient lists containing the surgeon’s indications of agreement were placed in opaque envelops and securely stored at an on campus location. At the end of the research period these patient lists were compiled in a database stored on a secure hospital computer in an anonymous fashion.

The primary outcome was the rate of agreement at the time of handover, expressed as a percentage of all handovers. Clinical disagreements were defined either as major or minor. A major disagreement was considered to be any disagreement event that fell into one of four pre-specified categories: (1) delay to operating room, (2) disagreement over diagnosis, (3) disagreement over operative technique, and (4) disagreement over disposition decisions. These categories were defined a priori. All other disagreements were considered minor. Post-hoc analysis of patient outcomes was explored by examining patients’ electronic records as well as morbidity and mortality records. Additional outcomes included rate of disagreement by age, gender, and disease type. Morbidity and mortality data were collected prospectively on a daily basis.

T-test, chi-square, and Mann-Whitney U tests were used based on data type to determine statistical significance within 95 % confidence. Statistics were calculated using SPSS Statistics version 20.0.0.

## Results

**Fig. 1:**
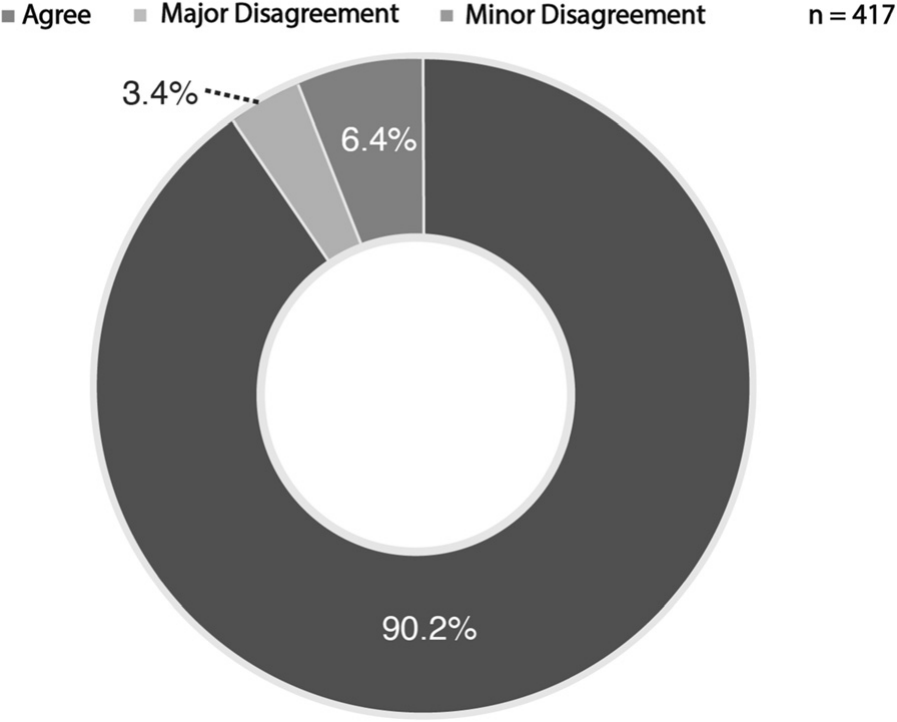
Proportion of clinical agreement at handover by agreement type

Receiving surgeons completed the required study patient list data for 55 of a possible 76 handover days resulting in a 72 % completion rate. A total of 417 unique patients were handed over during this period giving an average of 7.6 patients handed over daily. For the “Monday morning” handover days, (which the entire team was handed over to a new consultant of the week) there was an average of 13.1 patients handed over. For regular weekdays where another consultant may have been covering overnight, the average number of patients handed over was 5.5. Among the patients handed over, 41 disagreements were identified (Table 1). This represented an overall disagreement rate of 9.8 %. Of these disagreements, 15 were identified as major resulting in a major disagreement rate of 3.6 % (Fig. 1). There was a trend toward an increased frequency of minor disagreements (6.2 % minor vs. 3.6 % major); however, this did not reach statistical significance (*p* = 0.086).

**Table 1:** Handover agreement rate

Primary Outcome Category	Number	Rate
Total	417	-
Agree	376	90.2 %
Major Disagreement	15	3.4 %
Minor Disagreement	26	6.4 %

Of the patients handed over for which there was a major disagreement, 3 involved a delay to the operating room, 4 were the result of disagreement over diagnosis, 3 represented a disagreement over the operative technique, and 5 represented a disagreement over disposition (Table 2). Among the patients for whom there was a major disagreement, 33 % carried a diagnosis of large bowel obstruction and among patients for whom a minor disagreement was identified the most frequent diagnosis was small bowel obstruction at 23 %. However, none of the major disagreement categories were statistically over-represented compared to another.

**Table 2:** Disagreements by diagnosis and indication

Major Disagreement Diagnosis	Number
Small Bowel Obstruction	1
Large Bowel Obstruction	5
Trauma	0
Appendicitis	1
Biliary Disease	2
Other	6
Minor Disagreement Diagnosis	Number
Small Bowel Obstruction	6
Large Bowel Obstruction	2
Trauma	2
Appendicitis	2
Biliary Disease	2
Other	12
Reason for Major Disagreement	Number
Delay to OR	3
Wrong Diagnosis	4
Wrong Operative Technique	3
Disposition	5

**Fig. 2:**
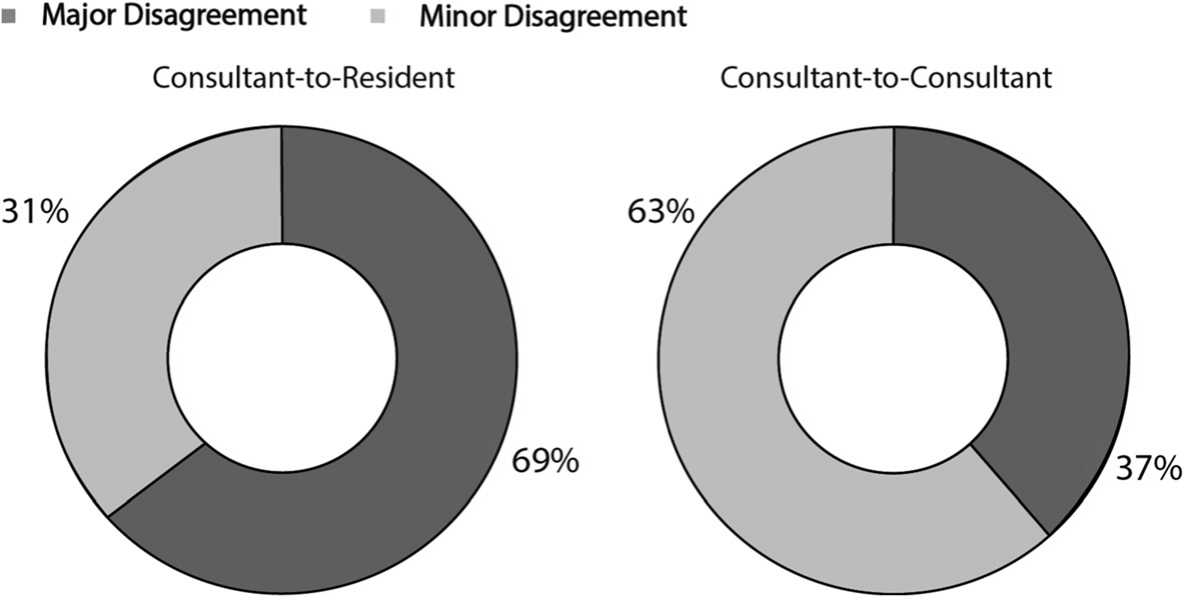
Proportion of disagreements by disagreement class

The level at which the disagreement occurred could be determined for 27 of 41 disagreements. Consultant to consultant disagreements were classified as major disagreements 63 % of the time and consultant to resident disagreements were major 31 % of the time (*P* = 0.217) (Fig. 2).

The mean age of patients for whom there was a disagreement was 63 years compared to 57 for those whom no disagreement was indicated (*p* < 0.05) (Table 3). Length of stay for those patients for whom there was a disagreement was an average of 3.5 days compared to 5.2 days for those whom no disagreement was identified (*p* = 0.649).

**Table 3:** Patient characteristics by agreement type

	Agree	Disagree	*P*
N	376	41	
Age (mean)	57	63	<0.05
Length of stay (mean)	5.2	3.5	=0.649

There were a total of 4 deaths among patients handed over for a mortality rate of 0.96 %. There were 25 morbidities identified resulting in a morbidity rate of 6.0 %. Among patients for whom there was a disagreement, there was one death leading to a mortality rate of 2.4 %. This compared to 3 deaths, and a mortality rate of 0.80 %, among patients for whom there was full agreement (*p* = 0.307). There were 4 morbidities among patients whom there was clinical disagreement giving a morbidity rate of 9.8 % compared to a morbidity rate of 5.6 % (*p* = 0.462) for patients for whom there was full agreement.

## Discussion and conclusions

Due to the diverse and complex nature of clinical decision making, the occasional clinical disagreement between providers is understood to be an expected feature of modern medical practice [14]. The wide range of training and experience embodied by the various providers involved in a patient’s hospital course inevitably leads to differences of opinion. In the traditional model of surgical care, a single surgeon was ultimately responsible for all aspects of patient care throughout the patient’s admission, and could dictate the overall trajectory of patient care [1]. The authority of a single surgeon over the patient’s course in this practice model made differences of opinion unlikely to have an impact on continuity of care [1]. However, this model of care is changing, and the emergence of acute care surgery teams has made multiple surgeons primarily responsible for a single patient’s care at different points during an admission. In such an environment, disagreements over clinical management have the potential to significantly impact patient care – positively or negatively. A recent evaluation of acute care surgery handover practices indicated that problematic handovers could have a negative impact on patient outcomes, and the experience of learners [15]. In that study residents felt, that on average, inadequate handover contributed to at least a minor harm in 2.7 individual patients, and a major harm in 0.6 individual patients over the course of their training [15]. With this evidence in mind, we feel that the clinical disagreements which occur when key patient care providers change over have the greatest potential to impact continuity of care, and ultimately affect patient outcomes.

Clinical disagreement among attending surgeons has previously been described in contexts other than patient handover. In one example, when evaluating the same group of patients to determine whether surgery for peptic ulcer disease had been effective, 2 senior surgeons agreed on the patient outcome less than two thirds of the time [16]. Despite the known occurrence of clinical disagreements and the potential implications these disagreements have on patient care continuity, until now no research has been conducted to determine the frequency to which such disagreements occur. We found an absolute clinical disagreement rate between surgeons at a single tertiary care institution to be 9.8 %. Since no previous research has attempted to estimate clinical disagreement under similar conditions, it is difficult to imply how this disagreement level might compare to other institutions. As such, we believe that this level of disagreement has the potential to represent a benchmark for the evaluation of handover practices of other academic institutions.

Of the 9.8 % of handovers identified as clinical disagreements, 3.4 % fell into the category of major disagreement. The pre-specified major disagreement categories (diagnosis, time to OR, disposition, and operative technique) were felt to represent 4 broad categories where a disagreement would most likely result in a change to the patient care plan. Some of the more common examples of minor disagreements included disagreements over specific diagnostic tests ordered, antibiotic choice, involvement of consulting medical services, and the timing of drain/tube removal. In many cases, no clearly established clinical guideline exists for these issues, and as a result the clinical choices surrounding the more frequent minor disagreements are influenced heavily by clinical judgment and experience.

Although not statistically significant, there was a trend toward consultant to consult disagreements more frequently being classified as major compared to consultant to resident disagreements (63 % vs 31 %). This potential difference is likely explained by the fact that in our institution the 4 major disagreement categories tended to represent consultant level decisions, whereas the more frequent minor disagreements tended to occur over issues which a resident may be encouraged to exercise some autonomy. Finally, we demonstrated a trend toward an increased mortality (2.4 % vs 0.8 %, *p* = 0.307) and increased morbidity (9.8 vs 5.6 %, *p* = 0.462) among patients for whom there was a disagreement. The intention of this study was to look at the thought process of surgeons during handover and not to evaluate patient outcomes; however, this trend certainly supports the authors’ belief that among patients in whom there is a clinical disagreement, there may be associated poor clinical outcomes. Also, patients in the disagreement group were older (57 vs 63) which may suggest that they have more complex conditions that could generate more clinical disagreements. We were unable to differentiate if indeed it was the more complex medical issue or the concerns with continuity of care that were responsible for differences in clinical outcomes. A larger prospective study would be required to determine that relationship.

The fact that clinical disagreements occurred between surgeons at handover also raises the question of the source of these disagreements. On this issue, the literature has previously demonstrated that the process of acquiring clinical information is subject to a number of influences, which may lead one clinician to have different information than another about the same patient [13]. A unique aspect of this research, is the fact that the surgeon (and the resident team) handing the patient over represents the source of the information being utilized by the receiving surgeon to make a clinical judgment [12].

Our study has several limitations. This study was limited to a single institution, and a relatively small number of surgeons (*n* = 6). In addition, these surgeons have worked closely together for a number of years in a teaching hospital environment; which, may have lead to relatively homogenous practice patterns, and consequently low levels of disagreement. As a result, it is reasonable to expect that larger institutions with a greater number of surgeons who have a wider variety of clinical experience and training may have higher disagreement rates.

It is also possible that the 9.8 % disagreement rate may underestimate the actual frequency of disagreements at our institution, since it relied on the careful consideration and effort of the receiving surgeon to indicate that a disagreement had actually occurred. It may have simply been easier for surgeons to indicate an agreement as opposed to offering a short written explanation, as was required of the study protocol, for all disagreements. Additionally, concern about the possibility of offending a clinical partner could have entered into the minds of participants potentially influencing their behavior despite assurances that no identifying data would be collected, and that participants were blinded to the responses of their colleagues. These factors could have potentially reduced the number of disagreements identified by participants increasing the risk of a type II error in this study.

This research sheds a unique light on clinical judgments at the time of handover by providing a unique estimate of clinical agreement between surgeons. With a 90.2 % agreement rate, this study demonstrates a high degree of concurrence among surgeons caring for patients in acute care surgical teams. Further research, with larger volumes, is required to elucidate the underlying reasons for the disagreements that do occur as well as to further elucidate the role of clinical disagreements on patient outcomes.
